# A Transfer of Technology from Engineering: Use of ROC Curves from Signal Detection Theory to Investigate Information Processing in the Brain during Sensory Difference Testing

**DOI:** 10.1111/j.1750-3841.2010.01863.x

**Published:** 2010-11

**Authors:** Sukanya Wichchukit, Michael O'Mahony

**Affiliations:** Author Wichchukit is with Dept. of Food Engineering, Faculty of Engineering at Kamphaeng Saen, Kasetsart Univ.Kamphaeng Saen Campus, 1 Malaiman, Kamphaeng Saen, Nakorn-pathom 73140, ThailandAuthor O'Mahony is with Dept. of Food Science and Technology, Univ. of CaliforniaDavis 1 Shields Avenue, Davis, CA 95616. Direct inquiries to author Wichchukit (E-mail: fengskw@ku.ac.th).

**Keywords:** A Not–A test, β-criterion, cognitive strategies, decision rules, response bias, ROC curves, same–different test, sensory difference tests, Signal Detection Theory, τ-criterion

## Abstract

This article reviews a beneficial effect of technology transfer from Electrical Engineering to Food Sensory Science. Specifically, it reviews the recent adoption in Food Sensory Science of the receiver operating characteristic (ROC) curve, a tool that is incorporated in the theory of signal detection. Its use allows the information processing that takes place in the brain during sensory difference testing to be studied and understood. The review deals with how Signal Detection Theory, also called Thurstonian modeling, led to the adoption of a more sophisticated way of analyzing the data from sensory difference tests, by introducing the signal-to-noise ratio, d′, as a fundamental measure of perceived small sensory differences. Generally, the method of computation of d′ is a simple matter for some of the better known difference tests like the triangle, duo–trio and 2-AFC. However, there are occasions when these tests are not appropriate and other tests like the same–different and the A Not–A test are more suitable. Yet, for these, it is necessary to understand how the brain processes information during the test before d′ can be computed. It is for this task that the ROC curve has a particular use.

## Introduction

Sensory difference or discrimination tests are important in food sensory science. They are used for determining whether judges can discriminate between products that are so similar that they can be described as confusable. Such tests are used for quality assurance, ingredient specification, product development, and studies of the effects of processing change, packaging change, and storage, as well as for various psychophysical measures. Sometimes they are used analytically with trained judges under controlled conditions and such tests then come under the general heading of what has been called Sensory Evaluation I (O'Mahony 1995a). At other times, they are used to study whether consumers perceive such differences under normal conditions of use (Sensory Evaluation II).

For sensory difference testing, food scientists adopted forced-choice tests (Peryam and Swartz 1950; Peryam 1958; Amerine and others 1965; Meilgaard and others 1991; Stone and Sidel 1993; Lawless and Heymann 1998; Kemp and others 2009) of which the triangle, duo–trio, 2-AFC, 3-AFC are probably the most well known. The data generated by these tests are generally analyzed to determine whether the differences measured are statistically significant or whether they could have occurred by chance. It should be remembered, however, that statistical significance depends not only on the size of the difference but also on the size of the sample. Such an analysis does not indicate the really important variable, the size of the difference. Obviously, the greater the difference, the greater the proportion of tests performed correctly. Yet, comparisons between the proportion of tests performed correctly for duo–trio and a triangle tests are complicated by their different chance probabilities. What is required is a fundamental measure of difference that is independent of the method used to measure it.

## Derivation of the Fundamental Measure, Signal Detection Theory, Thurstonian Modeling and d′

The required fundamental measure came from an unexpected source: electrical engineering and more specifically Signal Detection Theory, which is still an active and developing field (Hancock and Wintz 1966; Tuzlukov 2001; Barkat 2005; Levy 2008). The fundamental measure in question derived from Signal Detection Theory was the so called signal-to-noise ratio. It is worth pausing to consider how Signal Detection Theory was developed and to consider the meaning of the signal-to-noise ratio.

Part of Signal Detection Theory was concerned with discriminating input elicited by a target stimulus (signal) from background “noise.” By noise is meant “random and unpredicted signals produced by natural processes, both internal and external to the system. When such random variations are superimposed on an information bearing signal, the message may be partially corrupted or totally obliterated” (Carlson and others 2002). An important source of noise which is inherent internally in all electrical systems, is so-called “thermal” noise. This “internal” noise is caused by the random motion of charged particles in the hardware, usually electrons, generating random currents and voltages (Carlson and others 2002). Obviously, the stronger the intensity of the signal, the less likely it is to be obliterated by this randomly varying noise.

An application of Signal Detection Theory was in the development of radar (1938 to 1945) for detecting enemy aircraft during the Second World War (Carlson and others 2002). The radar system sends out and receives signals. One task is to distinguish the received signals from background noise. The greater the intensity of the signal compared to the intensity of the noise, the less will be the chance of the signal being obliterated. Thus, the ratio of the intensity of the signal to the intensity of the randomly varying noise, the so-called signal-to-noise ratio, is an all important measure (Porat 1997; Tuzlukov 2001).

This can be visualized by considering the noise as varying as a frequency distribution in a particular position on an intensity axis of electrical activity. This might be the result of thermal noise. Now consider a signal that has entered the system, perhaps indicating an enemy aircraft. It will be overlaid by the noise and can be then represented by a 2nd frequency distribution further up the axis. The distance between the 2 distributions represents the strength of the signal. The standard deviation of the frequency distribution represents the variation in intensity of the noise. The greater the distance between the 2 distributions, the greater the signal strength and the easier it will be to discriminate the signal from the noise. In Signal Detection Theory, the distance between the means of these 2 distributions is measured in units of standard deviations of the noise distribution. In other words, the strength of the signal is measured in units based on the variability of the noise sample. In this way, it is a signal-to-noise ratio. In general, how well a detection apparatus detects signals from the background noise is represented by this signal-to-noise ratio. Some systems may be more sensitive than others and so the signal distribution moves further up the axis and the signal-to-noise ratio will be greater. Yet, the exact method used to measure the distance between the 2 distributions and the variation of the noise distribution does not affect the signal-to-noise ratio. Thus, it is a fundamental measure just like molecular weight, voltage, chemical concentration, and so on.

A selection of the ideas and approaches in the Engineers’ version of Signal Detection Theory, were selected and transferred for the theoretical development of Sensory Psychophysics (Green and Swets 1966; McNicol 1972; Macmillan and Creelman 2005). The important signal-to-noise ratio was adopted and represented by the symbol d′, pronounced “dee prime.” In psychophysics, it can be argued that the simplest application of Signal Detection Theory was for judges to distinguish between sensory input from “threshold” levels of visual or auditory stimuli, from the sensory input associated with the absence of such stimulation. When these “threshold” visual or auditory stimuli were absent, the nerves in the nervous system would still fire spontaneously sending a randomly varying volley of signals to the brain. This internal “neural noise” in the nervous system can be treated as analogous to “thermal noise” in engineering systems. The task of the judge in Psychophysics was to distinguish between the signal (the presence of the appropriate threshold level stimulus) from the background neural noise.

Yet, this idea was soon extended to any pair of stimuli, of which one was designated the “signal” stimulus and the other the “noise” stimulus (Green and Swets 1966; McNicol 1972). For food sensory science the same approach can be taken for difference testing. The task of the judge is to distinguish between 2 very similar and confusable food samples. One is designated as the signal sample (in the simplest case it may have some added ingredients) and the other is designated as the “noise” sample (without the added ingredients). The task of the judge is to compare the 2 samples and to attempt to identify the sensations elicited by the added ingredients that distinguish the “signal” sample from the “noise” sample.

What makes Signal Detection Theory particularly appropriate for transfer to food sensory science is its treatment of variance. For radar, even though the physical signal might be constant, the signal that is processed by the receiving apparatus is not. There is a source of variance: thermal noise. For sensory difference testing of foods, as opposed to visual or auditory stimuli, the “internal” sources of variance come not only from the neural components of the sensory system but also from variation associated with the sensory receptors. For example, in the mouth, chemical taste stimuli released from the food are diluted by the ever varying salivary flow, while sensory adaptation attenuates the strength of the signal transmitted to the brain. This attenuation is, in turn, varied by movements of the food within the mouth. Difference tests usually require judges to make comparisons of the taste of a food sample currently in the mouth with the memories of prior tasted samples; these have variance. Furthermore, the food samples themselves may not be homogeneous providing an “external” source of variance or noise. Accordingly, as in Engineering, a food stimulus may be represented by a perceptual frequency distribution, describing the variance, along some form of perceptual intensity axis. The variance effects are generally small and would not be noticed while consuming foods but they become significant in difference testing, where judges focus on very small changes.

Signal Detection Theory is generally called Thurstonian Modeling in Food Science and it has been reviewed elsewhere by several researchers (O'Mahony and others 1994; O'Mahony 1995b; Rousseau 2001; O'Mahony and Rousseau 2002; Lee and O'Mahony 2004, 2007a) so it will only be briefly mentioned here. Experiments have shown that for the same pairs of stimuli, the same judges will perform better with some test protocols than others (for example: Stillman 1993; Tedja and others 1994; Stillman and Irwin 1995; Rousseau and O'Mahony 1997, 2000, 2001; Rousseau and others 1998, 1999; Dessirier and O'Mahony 1999; Lau and others 2004; Kim and others 2006; Lee and O'Mahony 2007b; Lee and Kim 2008; Lee and others 2009; Kim and others 2010). Several models have been developed to explain these differences (Lee and O'Mahony 2007a). Most of these models consider the effects of how physical interactions in the mouth (mentioned previously) and cognitive effects like cognitive contrasts and forgetting, affect the measured sensitivity of judges. They focus on what affects the signal-to-noise ratio (d′). Such models are Sequential Sensitivity Analysis (O'Mahony and Odbert 1985; O'Mahony and Goldstein 1987), the Conditional Stimulus model (Ennis and O'Mahony 1995), the Cognitive Contrast model (Lee and O'Mahony 2007b) and the most recent refinement, the Sequential Perception Analysis model (Lee and others 2009).

Thurstonian modeling takes a quite different approach. It considers how variations in the judge's decision rule or cognitive strategy involved in difference testing procedures, affects performance. In other words, it focuses on how the brain organizes the input from the senses when making fine discriminations. This approach does not consider factors that affect the signal-to-noise ratio (sensitivity, d′) per se. Instead, it considers how for a given sensitivity (d′), processing of the sensory input in the brain affects performance. Ura (1960) first applied Thurstonian ideas to 2-AFC, triangle and duo–trio tests. The Thurstonian/Signal Detection approach was further developed for both univariate (David and Trivedi 1962; Bradley 1963; Vessereau 1965; Frijters 1979a, 1979b, 1980, 1981a, 1981b; Ennis and others 1988a) and multivariate measures (Ennis and Mullen 1985, 1986a, 1992a, 1992b; Kapenga and others 1987; Mullen and Ennis 1987, 1991; Ennis and others 1988b; Mullen and others 1988; Ennis 1988a, 1988b, 1990, 1992). Based on such models, tables have been published for given tests protocols, that allow d′ values to be determined from the proportion of tests performed correctly (Elliott 1964; Hacker and Ratcliff 1979; Frijters and others 1980; Frijters 1982; Ennis and Mullen 1986b; Ennis 1993; Ennis and others 1998). For forced choice tests, d′ measures are gradually being adopted by the food, personal, and household products industries. The biggest barrier to adoption is getting a “feel” for d′. One way of doing this is to consider the 2-AFC test. The chance of guessing when judges cannot tell the difference between the 2 foods is 50%. Should the judges discriminate perfectly, the judges will get 100% of the tests correct. Half way between chance and guessing is 75%. A d′ value of unity is equivalent to 76% tests correct. It can be seen as the threshold value. A d′ of 1.5 yields 86% tests correct, a d′ of 2 yields 92%, and a d′ of 3 yields 98% correct. Perfect discrimination yields a d′ of infinity but then we are no longer in the area of difference testing; the stimuli are no longer confusable and d′ is inappropriate.

The traditional forced choice tests generally ask questions like which of 2 or more samples is more intense in some attributes or which of 3 food samples is different from the other 2. Because the judges are forced to make a choice, these tests resist what is called “response bias” (Lee and O'Mahony 2004), as will be discussed subsequently. Yet, other tests like the “same different” test and the “A Not –A” test are not. They are prone to such bias. To calculate d′ in these circumstances, it is important to know how the judges are processing information in their brains while they perform the tests. This is where another Engineering concept from Signal Detection Theory, the ROC curve (Dorf 1997; Hippenstiel 2002; Barkat 2005; Levy 2008) becomes useful. Yet, to understand this, it is first necessary to consider response bias.

## Response Bias, a Basic Problem for Difference Tests: Beta (β) and Tau (τ) Criteria

To understand response bias it is first necessary to understand the underlying questions that are implied during discrimination tests. Common sense questions, like “Are these two food samples the same or different?” are appropriate for easily discriminated stimuli where perceptual differences are large. However, for difference tests, where perceptual differences are so small as to make the stimuli confusable, such questions become biased. When differences get smaller new rules apply, just as when particles get smaller, common sense Newtonian mechanics no longer applies and is replaced by quantum mechanics.

Consider a judge having to discriminate between 2 confusable foods: “S” and “N.” He is presented with each food one at a time. His task is to identify whether each food is either “S” or “N.” In Signal Detection parlance, this task is called the Yes–No task or procedure (Green and Swets 1966; McNicol 1972). Because the foods are confusable, the decision whether the food is “S” or “N” will be difficult to make. The judge's response will depend on 2 things. First, it will depend on how well his sensory systems distinguish between the sensory input elicited by “S” and “N.” Second, it will depend on whether he feels the sensations elicited by “S” or “N” are more likely to have come from “S” or “N.” For example, when he tastes “S,” he has to decide whether the sensory input from “S” should be included in the “S” category or whether it would be better placed in the “N” category. It depends on where he “draws the line” in his perceptual continuum between “S” and “N” (Green and Swets 1966; O'Mahony 1992, 1995b; Rousseau 2001). The line is the border between his concepts of “S” and “N.” Depending on where he draws the line, he will be more inclined or biased to categorize his sensations as “S” or more inclined to categorize them as “N”: hence the term “response bias.” The line has a technical name, it is called the β-criterion.

It can be seen that response bias is a problem for the Yes–No procedure. Consider a judge presented with a sample of the food “S.” Even though the judge's sensory system may correctly distinguish the sensory input elicited by “S,” he may not wish to say it was “S” because he drew his line, the β- criterion, in the wrong place. He wrongly categorized that sensation as more typical of “N.” His β-criterion was in an inappropriate position so that the sensations elicited by “S” fell on the “N” side of the line. The problem is that the β-criterion is not stable. For a judge, it will vary over time and it certainly varies between judges. Therefore, because of the instability of the β-criterion, a judge being tested using the Yes–No procedure, may receive the sensory input elicited by a food quite clearly but give an incorrect response because of a wrongly placed β-criterion on his perceptual continuum.

Besides the β-criterion, there is another type of criterion called the τ-criterion (Rousseau 2001; O'Mahony and Rousseau 2002; O'Mahony and Hautus 2008). The τ-criterion is concerned with how different 2 foods need to be to be reported as “different.” It can be visualized as a sensory yardstick. If the sensations elicited by the 2 foods are more different than the length of the yardstick, the foods will be reported as “different.” If they are not, they will be reported as “same” (Irwin and others 1993; Irwin and Francis 1995; Rousseau and others 1998; Rousseau 2001). As with the β-criterion, the τ-criterion is unstable and for a given judge, will vary over time and as well as varying between judges. Therefore, because of uncontrolled nature of the τ-criterion, a judge being asked whether 2 stimuli are the same or different, may receive the correct sensory input elicited by the foods in question but respond incorrectly because of the inappropriate length of the τ-criterion in his perceptual continuum.

Computations based on Signal Detection Theory are used to circumvent these problems (Green and Swets 1966; O'Mahony and Rousseau 2002; Macmillan and Creelman 2005). Yet, for the same–different and A Not–A tests, such computations require a knowledge of how the sensory input arriving at the brain is organized, when the judge is making his decisions during the sensory testing procedure. Is it organized using a β-criterion (β-cognitive strategy) or using a τ-criterion (τ-cognitive strategy)? The ROC curve (Dorf 1997; Hippenstiel 2002; Barkat 2005; Levy 2008) derived from Signal Detection Theory, provides a way of finding out.

## Hits, False Alarms, and Constructing an ROC Curve

In a situation where a sensory difference test has response bias, an ROC curve can do 2 things. It can provide one of the various methods of computing d′. Also, should it be necessary, distortions in the curve can also provide insight into how the brain processes information during the testing procedure. This is a novel use that was not envisioned by the engineering community. Consider a judge being tested by the Yes–No procedure (Green and Swets 1966). A series of 2 confusable foods, “S” and “N,” are presented to the judge in random order. The task for the judge is to identify which of these stimuli are “S” and which are “N.” Obviously, the procedure has inherent response bias. Should a judge be able to distinguish between the 2 confusable stimuli, his responses will depend on the position of his β-criterion. To circumvent this problem, it is necessary to apply Signal Detection Theory.

The trick used by Signal Detection Theory, involves not only recording whether a judge's response was right or wrong but also recording how the response was right or wrong. Imagine food “S” was slightly more rancid than “N” but the 2 were still confusable. The judge's task would be to identify “S” by detecting a slight rancidity signal and “N” by its absence of that signal. There are 4 possible outcomes. If the judge correctly identifies the rancidity of “S” and reports food “S” as being “S,” this is called a “Hit.” If the judge identifies “N” as “S,” imagining he could taste rancidity that was not there, it is called a “False Alarm.” If the judge correctly identified “N” as “N,” correctly noticing the absence of rancidity, it is called a “Correct Rejection.” If the judge missed identifying the rancid taste in “S” and identified it as “N,” this is called a “Miss.” This more detailed analysis of the judge's responses, frees the data from the biasing effect of the β-criterion and enables a computation of d′. There are various ways such a computation may be performed but here the computation using the ROC curve will be described. This has the advantage that it has a built in check to determine whether the assumptions required for d′, mentioned previously, are upheld. The assumptions are that the perceptual distributions are normal with equal variance.

**Figure 1:**
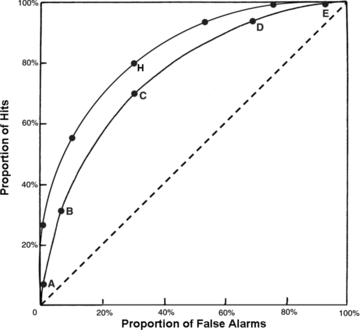
Two ROC curves representing different signal strengths.

An ROC curve is obtained when the proportions of hits is plotted against the corresponding proportion of false alarms, for various β-criteria. ROC curves have been reviewed elsewhere (Hautus and others 2008; O'Mahony and Hautus 2008). Such curves are illustrated in Figure 1. In the figure, consider point “C.” This is the result of a judge's attempts to identify correctly “S” and “N” by the presence or absence of the rancidity signal. He has 68% hits and 30% false alarms. Now, imagine that the experimenter manipulates the experimental situation so that the judge is more willing to say he can detect the rancidity (changes to a less strict β-criterion). This may be done by altering the probability of occurrence of “S” and “N.” Being more willing to report the presence of rancidity, he will then have more hits and false alarms. Depending on the strength of this effect, this could give him points “D” and “E” on the graph. Now assume that the experimenter manipulates the experimental situation to make the judge less willing to report the presence of rancidity, a change to a stricter β-criterion. This would result in fewer hits and false alarms. Again, depending on the strength of this effect, it could give points “A” and “B.” More manipulations can be made and more points obtained through which a curve can be drawn. This is the ROC curve.

If the intensity of rancidity in “S” was slightly greater, meaning that the signal strength for rancidity was increased, the judge would have more hits (say, 80% rather than 68%) for the previous number (30%) of false alarms (see point “H” directly above point “C”). In other words, when the judge reported that he was tasting “S,” the likelihood that he was actually tasting “S” (scoring a hit) would be increased. In the same way, a whole new set of points would describe a second ROC curve. For this greater signal strength (higher d′) it can be seen that the curve bows out further. It is possible from the degree to which the curve bows out to compute d′. This can be done by scanning the curve and feeding the data into an appropriate computer program. However, there are simpler computations that involve plotting various functions of the proportion of hits against the proportion of false alarms. For example, plotting z-scores associated with proportions of hits and false alarms, produces linear ROC plots. If the assumptions of normal distributions with equal variance hold, the plot will be linear with a slope of unity. Where the plot intersects the Y-axis gives the value of d′.

**Figure 2:**
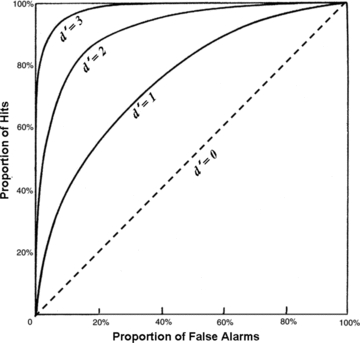
ROC curves for different d′ values.

Figure 2 illustrates a family of ROC curves with their corresponding d′ values. The more the curves bow out, the higher the value of d′. Should the judge be able to detect the difference between the 2 stimuli perfectly, he would have 100% hits and no false alarms. His ROC “curve” would be a dot in the top left hand corner of the figure. In this case, the stimuli would no longer be confusable and difference testing would not be appropriate. Note that the diagonal corresponds to a d′ value of zero. This is because if a judge cannot detect the rancidity signal, then when he reports that he can, the probability that it is actually the rancid food “S” or the nonrancid food “N” is equal. Accordingly, the proportions of hits and false alarms will be equal.

The problem with the ROC curve is the time required to construct it. Each point on the curve requires a separate Yes–No experiment for each β-criterion. However, a more efficient approach is to require the judges to work with several β-criteria simultaneously. Consider the detection of rancidity experiment. If a judge tasted “S” and reported that he detected rancidity (a hit), he could be asked if he were “absolutely sure,”“maybe sure,” or “only guessing.” Being absolutely sure he detected rancidity is equivalent to using a strict criterion; it would not happen very much (few hits, equivalent to say, point B in Figure 1). Being “maybe sure” is equivalent to using a less strict criterion (maybe point C in Figure 1). Feeling that he was just guessing is equivalent to using a very slack criterion (maybe point D).

Thus, adding sureness judgements can speed up the construction of the curve. The judge would then have 6 possible responses: “S” or “N” (rancid or not) qualified by “sure,”“maybe,” or “guessing.” The words used can be varied according to the judge (for example, “easy to detect”“difficult to detect,”“had to guess”). The important thing is to obtain graded responses to represent different β-criteria. This modification of the Yes–No procedure has been called the rating procedure (Green and Swets 1966) or the “rating scale task” (McNicol 1972). Sometimes a judge might not be able to deal with 6 categories, in which case it could be reduced to 4 (but no further) because there will be too few points to get a good representation of the curve. Note that a “don't know” response is not allowed; this task forces a choice of either “S” or “N.”

**Figure 3:**
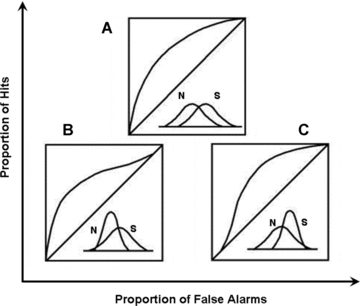
Distortions of the ROC curve caused by unequal standard deviations of the 2 perceptual distributions for foods “S” and “N.”

If the ROC curve (using the β-criterion) obtained from the data is symmetrical, it indicates that the perceptual distributions for the “S” and “N” foods are normal with equal standard deviations. This is a convenient way of checking that the assumptions for the computation of d′ are upheld. If the standard deviations of the normal distributions are not equal, the curves lose their symmetry (Hautus and others 2008; O'Mahony and Hautus 2008). The distortions can be seen in Figure 3. Looking at part (A), it can be seen that the distributions for “N” and “S” have the same standard deviations and the resulting ROC curve is symmetrical. In contrast, parts (B) and (C) indicate the situations where the standard deviation of “S” is greater than “N” or is less than “N,” respectively. The resulting ROC curves lose their symmetry in opposite directions. The variation in standard deviations could be caused by the rancidity in “S” affecting the variability of the sensory input elicited by that food. It could either increase variability (B) or decrease it (C). Thus, it can be seen that the shape of the ROC curve not only provides a way of computing d′ but it can also provide useful diagnostics.

**Figure 4:**
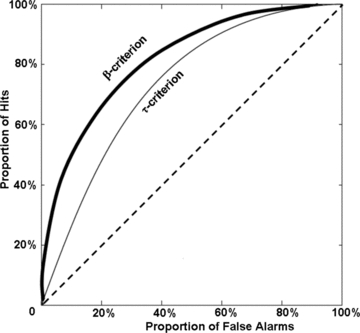
ROC curves for same–different judgments using either the β-criterion or the τ-criterion for the same d′.

So far, the discussion has been concerned with curves generated for a cognitive strategy using only a β-criterion. The distortions in the curves are caused by different variances for the signal and noise perceptual distributions. However, distortion of the ROC curve can occur for a quite different reason. With the same–different test, a τ-criterion might be used rather than a β-criterion. This can also cause a distortion causing the curve to lose its symmetry. Yet, this distortion is quite different to the distortion caused for a β-criterion when the variances are not equal. The resulting loss of symmetry of ROC curve caused by the adoption of a τ-criterion is illustrated in Figure 4. This will be discussed in a later section.

If the curves are not symmetrical, the assumptions required for d′ are broken. An alternative measure is required. One such measure is P(A) (Green and Swets 1966; McNicol 1972). This is simply the proportion of area under the ROC curve. The more sensitive the judge is to the rancid taste (the more he can distinguish “S” from “N”) the more the curve bows out and the greater is P(A). This measure can be used whether the curve is symmetrical or not. There are no assumptions required for P(A); it is a nonparametric measure. The interesting thing is that P(A) also corresponds to the estimated probability of correctly performing a 2-AFC test (Green and Swets 1966). This can be seen to be apparent from Figure 2. If a judge cannot distinguish between 2 stimuli, he will perform at chance levels. For a 2-AFC the estimated chance level is 50%. The proportion of area beneath the diagonal ROC for a d′ of zero is also 50%. For perfect discrimination, P(A) is 100% (all hits, no false alarms) as is the estimated proportion of correct responses for the 2-AFC. For a d′ value of unity, the estimated proportion of correct responses for a 2-AFC will be 76%, and this corresponds to the proportion of area under the ROC curve. Another name for the estimated proportion of correct responses in a 2-AFC test is John Brown's R-Index (Brown 1974; O'Mahony 1983, 1992; Lee and van Hout 2009). Thus, the R-Index and P(A) are the same; the R-Index belongs to the family of signal detection measures. Thus, from Figure 2, it can be seen that as d′ increases so does the R-Index, but it is not a linear relationship. Also, from Figure 4, it can be seen for the same–different test that responses using a β-criterion will produce a higher R-Index or P(A) than using a τ-criterion.

Despite the time required, ROC curves have been applied, albeit rarely, to food and beverage stimuli: Stull and others (1974) for ice cream; Owen and Machamer (1979) for wine and Paredes-Olay and others (2010) for olive oil. All studies were more concerned with areas under their ROC curves and did not compute d′ values.

## The Use of ROC Curves for Investigating the Decision Rules Used in Sensory Difference Tests: Same– Different Tests

In Psychophysics, the data obtained from Yes–No (or rating) procedures elicit symmetrical ROC curves, indicating the use of a β-criterion (Green and Swets 1966; O'Mahony and Hautus 2008). However, because of the time required, the Yes–No procedure is not generally used in Food Sensory Science. Yet, the same–different and A Not–A procedures are used. However, both should be approached with caution.

First consider the same–different test (Macmillan and Creelman 2005; Bi 2006; Kemp and others 2009) where a judge first tastes a standard stimulus and then tastes a comparison stimulus, which may or may not be the same as the standard. The task for the judge is to say whether the comparison is the same as or different from the standard. For this test, the exact protocol has not been standardized. There is a short version, where only 1 pair of stimuli is presented. There is also a long version, where 2 pairs are presented, 1 the same and the other different, although the judge is unaware of this. Bi (2006) indicated that the short version is statistically less powerful than the triangle or duo–trio methods, while Ennis (2004) indicated that the long version is more so. Rousseau and others (1998) using vanilla flavored yogurts, confirmed this. To add to the confusion, the test has also been described in the literature under a variety of other names (Pfaffmann 1954; Amerine and others 1965; Kaplan and others 1978; Meilgaard and others 1991; Stone and Sidel 1993; Lawless and Heymann 1998).

Logically, the same–different test could use a τ- or a β-cognitive strategy. For a τ-cognitive strategy, a judge whose senses have discriminated between 2 confusable stimuli, will report them as “same” or “different,” depending on whether the difference in sensory input exceeds his τ-criterion (different) or not (same). For a β-cognitive strategy, judges would arrange their perceptual space into 2 areas, divided by a β-criterion. They would then judge the sensations elicited by 2 stimuli as same or different, depending on whether they fell on the same side or different sides of the β-criterion dividing line. Essentially, judges would be making independent classifications of the stimuli, as opposed to making relative judgements as with the τ-criterion (Hautus and others 1994, 2009).

As long as graded responses are available (for example, “sure” not sure” and so on), ROC curves constructed from the data generated by same–different tests can be fitted by theoretical ROC curves, generated assuming τ- and β-cognitive strategies. (2 ROC curves generated for the same–different test using τ- and β-cognitive strategies, for the same d′ are illustrated in Figure 4). Maximum likelihood estimation can be used to see which gives the better fit (Hautus and others 1994). Goodness of fit is described by χ^2^ values and probability (p) values. The χ^2^ values indicate how different the data are from the fitted curves (using τ- against β-strategies). Therefore, smaller χ^2^ values indicate the strategy which is the better fit to the data. In the same way, the probability (p) values indicate the probability that the data can be explained by the model. Thus, higher *P* values indicate a better fit. One looks for lower χ^2^ and higher “*P*” values.

For fitting same–different ROC curves to the data, Lee and others (2007a) indicated that it would be most advantageous if d′ values for those data fell in the range of 1.8 to 2.5. ROC curves generated by such data would bow out to the right extent to facilitate determining whether its shape had a better fit to a β- or τ-strategy. This can be appreciated by a glance at Figure 4. Santosa and others (2010) later extended this range slightly to 1.75 to 2.6 and even used analyses for cases where d′ values were merely greater than threshold (d′≥ 1).

Regarding same–different tests for taste and food stimuli, Irwin and others (1993) demonstrated how ROC curves, derived from same–different tests for orange drinks, were best fitted assuming a cognitive strategy that used a τ-criterion. The same result was obtained by Stillman and Irwin (1995) using raspberry flavored drinks. Hautus and Irwin (1995) used same–different tests to distinguish between milks of different fat content. ROC curves were fitted to the data and despite some difficulty with the curves, the data supported the use of a τ-strategy. Use of the τ-strategy was also supported in the same laboratory by same–different experiments with auditory stimuli (Hautus and others 1994). Thus overall, studies support the notion of a τ-cognitive strategy for the same–different test.

Yet, Lee and others (2007a) wondered whether judges could be induced to use a β-criterion, if they had been categorizing the stimuli beforehand. They required judges to perform same–different tests using “threshold” concentration NaCl and purified water. Before performing the same–different tests with these stimuli, judges were required to assess separately whether the standard stimulus and then the comparison stimulus were NaCl or water, using the required graded responses. They were then required to assess whether the 2 stimuli tasted the same or different, again giving graded responses. It should be noted that these 2 sets of judgments were not necessarily consistent; a judge could assess 2 samples as NaCl but declare that they did not taste the same. To encourage further establishment of a β-criterion, judges were also given a prior formal warm-up (Meta-Garcia and others 2007) before testing. ROC curves indicated that 2 out of 4 judges then used a β-strategy in their same–different tests, while the other 2 used a τ-strategy. Thus, with taste stimuli, although a τ-strategy is the general rule, if judges were forced to categorize the stimuli beforehand, a β-strategy was seen to be possible.

This encouraged Santosa and others (2010) to question whether repeated use of the same–different test, with the same stimuli, might induce judges to become so familiar with those stimuli that they would begin to categorize them independently, so adopting a strategy with a β-criterion. Yet, after performing over 2000 tests, with threshold NaCl and purified water stimuli, ROC curves indicated that none of the 4 judges switched. They consistently used the τ-strategy. It would seem as though the results of Lee and others (2007a) were an intriguingly rare exception. Interestingly, 3 of the 4 judges reported using a τ-strategy, although one thought she also used a β-strategy, despite her data. A 4th judge claimed to be using a completely different strategy, despite his ROC curves indicating consistent use of a τ-strategy. He claimed to have learned the 4 possible sensation changes induced by the stimulus pairs and was categorizing these. This could be called a “β-strategy for stimulus pairs.” Perhaps his subjective reports were mistaken or perhaps this strategy produces an ROC curve that cannot, with present models, be distinguished from a τ-strategy ROC curve.

Yet, the results of Lee and others (2007a) were not a rare exception. Chae and others (2010), using milk stimuli, had consumers perform same–different tests after performing prior familiarization procedures to vary their state of mind. For this, 1 group used rank-rating to evaluate samples for liking and other integrated semantic attributes like freshness, well-being, and off-flavor. This was to cause them to perform the same–different test under an affective and hedonic state of mind, which was hypothesized to approximate more towards realistic conditions of consumption. A 2nd group used a familiarization procedure to put them in a more analytic state of mind, with rank-rate for similarity to a reference standard. The 1st group showed better discrimination, while ROC curves indicated that the data for both groups were best fitted by curves assuming a cognitive strategy that used a β-criterion. It would seem that specific activities performed prior to a same–different test were more efficacious at inducing a β-strategy than mere repetition of the test.

## The Use of ROC Curves for Investigating the Decision Rules for A Not–A Tests

Next, consider the A Not–A test which was described by Peryam (1958). It also has no fixed protocol. For one version, a standard stimulus (call it “A”) is given to the judges, who may taste it as often as required to become familiar with its flavor. Then a series of comparison stimuli are presented to the judge in random order. Some are the same as the standard (A), while others are the stimuli to be discriminated from the standard (Not-A). Judges are required to categorize which are which. In another version of the test, both stimuli (“A” and “Not-A”) are presented as references before the test. “Not-A” might be one stimulus or several. For these protocols, once judges have started tasting the comparison stimuli, the standard(s) cannot be retasted. Yet, Peryam (1958) and Pfaffmann (1954) suggested that the standard might be retasted occasionally as a reminder. Another version of the test presents “A” before every comparison as a reminder and this protocol is called an “A Not–A with reminder” (A Not–AR) (Lee and others 2007c). Versions of most of these protocols are described in standard texts (for example, Amerine and others 1965; A.S.T.M. 1968; I.S.O. 1987; Meilgaard and others 1991; Kemp and others 2009).

There are various ways of conceptualizing the “A Not–A” test and they lead to different assumptions about the cognitive strategies used. First, it could be conceptualized as an extended “same–different” test, where “A” and “Not-A” judgments are analogous to judgments of “same as” or “different from” the gradually failing memory of the standard(s). In this case, it would be modeled in the same way as the same–different test. Reminders would be seen as the opportunity of retasting the standard so that subsequent comparisons could be compared to the fresh memory of the reminder. The A Not–AR would merely be a succession of same–different tests. As with the same–different test it would be expected that ROC curves derived from such an analysis would best be fitted by curves using a τ-cognitive strategy with the possibility of a β-cognitive strategy.

Santosa and others (2010) required judges to perform A Not–A tests immediately after performing over 2000 same–different tests (described previously). The same stimuli were used as in the same–different tests described above and 5 comparison stimuli were tasted after tasting the standard (a random order of “A” and “Not-A”). One goal of her study was to see whether the prior same–different tests might induce judges to compare the comparison stimuli to the stimulus tasted immediately beforehand rather than to the standard stimulus (“A”). Accordingly, she analyzed her ROC curves assuming that the A Not–A test was an extended same–different test. In her study, 2 of the judges appeared to continue using the τ-strategy they had used in the same–different test. Yet, 1 judge who had steadfastly used the τ-strategy throughout 2130 same–different tests, continued to do so for 650 A Not–A tests. Then, ROC curves indicated that she had started to use a β-strategy, as had been hypothesized for the effect of repetition on same–different tests. Yet, the effect of mere repetition on inducing a β-cognitive strategy could hardly be said to be immediate. Regarding the effects of prior same–different testing, all judges reported that at first, their cognitive strategy for the A Not–A test was affected by the prior same–different tests. They reported that instead of comparing the 5 comparison stimuli with the standard, they compared them with the immediately preceding stimulus. Yet, this did not show up in the ROC data, except for 1 judge. He used a τ-strategy for such comparisons. After that, he switched mostly (but not always) to comparisons with the standard. Yet, this was slight evidence for a strategy whereby initially the comparison stimuli were not compared to the standard stimulus but to immediately preceding stimuli. This might be called a “successive same–different strategy.”

The 2nd way of conceptualizing the A Not–A method is quite different. Hautus and others (2009), conceptualize it as equivalent to the signal detection Yes–No procedure, which also uses single presentations of stimuli. For example, using the Yes–No procedure, a judge might be reporting whether for single presentations, the tongue is experiencing a taste or not (O'Mahony 1972a, 1972b). Similarly, the A Not–A requires the judge to report, for single presentations, whether the judge is experiencing the taste of “A” or not. Being equivalent to the Yes–No procedure, the A Not–A is modeled using a β-strategy (Green and Swets 1966; Macmillan and Creelman 2005; Hautus and others 2009). There is no equivalent model for the Yes–No procedure with a τ-strategy.

This β-strategy argument also applies to the A Not–AR protocol, where a reminder is presented before each comparison. The reminder is not conceptualized as a stimulus for comparison as in a same–different test. The purpose of the reminder is merely to evoke the firmly established memory of “A” already embedded in the memory system, with which the comparison stimuli are being compared. Because the reminder is not a stimulus for comparison, the actual comparison stimuli can be seen as being presented singly as in the A Not–A test and thus a β-cognitive strategy is conceptualized.

Yet, Hautus and others (2009) did consider the possibility in the A Not–AR method, that the comparison stimuli might be compared with the reminders in a way similar to a series of same–different tests using a τ-criterion. They go on to explain that in this case, estimated performance would be reduced by a factor of 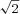. Then considering the A Not–A itself, the comparisons would be made with the fading and distorting memory of the initially presented standard (“A”). In this case, because of the time lapse between tasting “A” and the comparison stimuli, performance might be reduced by a factor greater than 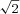.

Why are there contradictory ways of conceptualizing the A Not–A method? It would seem to depend on assumptions about which memory exemplars the comparison stimuli are being referred to. Are comparisons made to firmly established stable exemplars, perhaps in the long-term memory system (β-strategy)? Or are the comparison stimuli being referred to the fading and distorting temporary exemplar(s) generated by the initially presented standard (A) or standards (“A” and “Not-A”).

For the Yes–No procedure, it is assumed that judgments are made relative to firmly established stable exemplars embedded in the memory. They are not made relative to fading and distorting temporary exemplars generated by some prior tasted stimuli. For example, in a detection experiment when a judge decides whether the tongue is experiencing a taste or not (O'Mahony 1972a, 1972b) the exemplars for the presence of a taste or its absence are firmly established and embedded in the long-term memory. They are expected to persist outside the confines of the experiment; people do not forget the difference between a “taste” and “no taste.” Thus, the single presentations of the comparison stimuli will elicit a β-strategy.

Yet, in an A Not–A test, it is reasonable to question whether prior presentation of the standard stimulus (or stimuli) is sufficient to embed stable, firmly established exemplars in memory, so that they can be referred to in the same way as memories of the sensations like “taste” against “no taste,”“light” against “dark,” and “sound” against “silence”? If they can, then conceptualizing the A Not–A as a Yes–No procedure is certainly justified. If not, it is then likely that the comparison stimuli will be referred to the more temporary and distorting memory exemplars of the prior presented standard stimulus or stimuli (“A,”“Not-A”) or any later presented reminder stimuli. In this case, a same–different model could be applied to these comparisons and a τ-cognitive strategy might be expected. These questions are important for establishing the assumptions required for calculating d′ for this method. Yet, they are experimental questions and once again the ROC curve becomes a useful tool.

Lee and others (2007b) compared performance on A Not–A tests with ranking. Using 6 margarine products, panelists experienced with margarine tasting, performed a ranking test and the A Not–A test, using 2 protocols. For the 1st protocol, only a single standard (A) was presented beforehand, although it could be retasted as a reminder during testing as often as desired. For the 2nd, all 6 products were presented as standards beforehand, 1 for “A” and 5 for “Not-A.” However, these could not be retasted during testing. R-Index values, equivalent to the proportion of area under the ROC curve (Brown 1974; O'Mahony 1983, 1992; Lee and van Hout 2009) were calculated as measures of performance.

Ranking gave higher R-Index values than either version of the A Not–A test, probably due to the forced choice nature of the task with a consequent elimination of boundary variance. The A Not–A, the protocol, where all samples were presented as standards beforehand, elicited the better performance of the two. For explanation, they argued that when only “A” was presented initially, the concept of “A” induced could have been too broad and could have included some of the Not-A stimuli, causing errors of identification. Yet, when all stimuli were presented initially, judges would have had more chance of defining the boundaries of the “A” concept and establishing a β-criterion boundary between “A” and Not-A” stimuli. This would have produced better performance. They also surmised that the prior tasting of standards might have had some elements of a “warm-up” (Meta-Garcia and others 2007), which would have assisted with the establishment of a β-criterion. They entertained a further possibility. When only “A” was presented as the standard, with not enough information available to construct a β-criterion, judges might have had to refer the comparison stimuli to the distorting temporary memory exemplar for the prior presented standard stimulus (A). In doing so, they would probably have used a τ-criterion. They hypothesized that the inferior performance when using the τ-criterion might be due to its possible instability. Yet, inspection of Figure 4, illustrates that at least for the same–different test, superior performance would be expected with a β-criterion (all standards presented) because the proportion of area under the ROC curve (R-Index) is always greater for a β-curve than a τ-curve (Irwin and Francis 1995). It is no coincidence that in psychophysics, the β-cognitive strategy has been called the “optimal strategy” or optimal decision rule (Noreen 1981; Irwin and Francis 1995; Dai and others 1996). The idea that 1 standard (A) might induce a τ-strategy, while more standards might induce a β-strategy, requires more examination.

The judges were panelists who were familiar with margarine testing. Their experience on margarine panels ranged 5 to 12 y. For some of the judges, the difference in R-Index values between the 2 A Not–A protocols was comparatively small. It is possible that they already had a set of exemplars in their memory, some of which might have been relevant to the A Not–A tests at hand.

Yet, Lee and others (2007c) reexamined the A Not–A test using only 2 margarine products and judges who were not experienced with margarine. They used a selection of 3 A Not–A protocols, two 2-AFC protocols and a same–different test. They used an A Not–A test with both standards (“A” and “Not-A”) presented beforehand for familiarization, followed by 6 comparison stimuli (3A, 3B) with no retasting of the standards. Similarly, they used the A Not–AR test with only “A” presented beforehand. They also used the A Not–A with voluntary reminders and only “A” presented beforehand. For all A Not–A tests, judges responded with sureness ratings for each comparison stimulus. They used 2-AFC tests where beforehand the 2 stimuli were presented for familiarization and then presented as a series of 2-AFC tests. After each 2-AFC, the individual stimuli were given sureness ratings. For a second 2-AFC, (2-AFC reminder) just 1 stimulus was given as a standard beforehand for familiarization and pairs of samples were presented for 2-AFC tests, followed by sureness rating for each stimulus, as previously mentioned. The difference here was that before each 2-AFC, the judges were given “A” as a reminder. Finally, judges were given same–different tests (short version) also with sureness ratings. Both stimuli were presented beforehand for familiarization followed by the same–different test; sureness ratings were not used.

R-Index (proportion of area under the ROC curve) and, where possible, d′ values were calculated. From these, the cognitive strategies used in each protocol were surmised. The researchers regarded the A Not–A test as a version of the standard Yes–No task with a corresponding β-criterion. For the A Not–A test where both standards were presented beforehand, R-Indices were higher than when only one standard was presented. Presenting both standards would facilitate formation of a β-criterion, resulting in superior performance, while one standard would not. The A Not–AR elicited higher R-Indices than when the reminder was voluntary. At first sight, this could be explained by constant reminders producing a better evocation of the firm memory of “A” in the A Not–AR test. Yet, the researchers reported that in the A Not–A voluntary reminder test, judges tended to taste the reminders more than in the A Not–AR. The researchers hypothesized that, given this, the lower d′ values for the A Not–A voluntary reminder could be explained differently by a lowering of sensitivity caused by more significant carry-over effects, caused by more frequent tasting of the reminder stimuli.

The possibility was considered that the presentation of only a single standard and the judges not being experienced with margarine products might hinder the establishment of a satisfactory β-criterion. Judges would then have had to resort to comparisons with the single standard and reminders. The more frequent the reminders, the better the performance. Again, such same–different relative comparisons would tend to use a τ-criterion and performance would be expected to be reduced.

Lee and others also computed values of d′ by fitting ROC curves for both β- and τ-models, except for the A Not–A test, where a τ-model is not available. They proposed that the d′ estimates for the A Not–A test (β-strategy) and the 2-AFC were not too dissimilar (1.55, 1.31, respectively) and could provide a reference level for comparison with the other protocols. Incidentally, d′ values derived from β- and τ-versions of the 2-AFC are the same because their criteria are optimized and stabilized by the instructions (O'Mahony 1995b; O'Mahony and Rousseau 2002). Should this stabilization not succeed because judges do not follow the instructions properly, then d′ values will be depressed.

For the A Not–A reminder task, the d′ value using a τ-model was closer to the reference level (1.43) than using a β-model (1.01). This suggested that the judges were tending not to use the reminder stimuli as mere reminders but rather as standards for comparison. For the A Not–A with voluntary reminders, the protocol has not yet been effectively modeled. Both d′ values were below the reference levels although the τ-model was closer, supporting a weak conclusion that the judges may have been resorting to comparisons with the reminders, using a τ-criterion as in the A Not–AR.

For the 2-AFC reminder, d′ values were depressed (0.81). The researchers suggested that the task was difficult because of the increased memory load. An alternative explanation is that the judges were not comparing the 2 stimuli relative to each other, as is required in the 2-AFC, but instead they were comparing them in terms of their similarity to the reminder stimulus. It can be argued that presentation of only a single standard would encourage this. It would then seem that they were performing duo–trio tests. Had d′ values been computed according to the duo–trio model (Ennis 1993), the value of d′ would have been considerably larger (1.81). This is rather high, suggesting that not all judges were using a duo–trio. For the same–different test, the d′ value for the β-strategy came closer to the reference levels, suggesting its use.

The final 2 experiments previously mentioned were interesting first looks at the various versions of the A Not–A tests. Some results were expected and some were a surprise. They suggest that the protocols with one standard (A) presented prior to the comparison stimuli might need to be treated as entirely different from protocols where 2 or more (A, Not-A) standards are presented. They also encourage some opposing assumptions. Further research is needed and the ROC curve, either by its shape or by the proportion of area beneath it (R-Index) will continue to prove a useful tool in such investigations.

## Conclusions

The use of ROC curves in Food Sensory Science is just beginning. Previously, this had been the domain of psychophysicists, often with visual or auditory stimuli. Yet, Food Sensory Scientists, using ROC curves to investigate cognitive strategies with taste or food stimuli, have produced some surprising results. The surprises may be due to the fact that most prior experiments come from the discipline of psychology using visual or auditory stimuli. Performing with these, judges can be envisioned as very highly experienced experts, having concentrated on them and communicated about them for most of their life. This is rarely true for taste and food stimuli. Unlike many other species, we rely mainly on vision and are not primarily guided by the chemical senses.

The same–different test has always been assumed to induce a τ-cognitive strategy. There had been 2 exceptions where same–different tests using kanji (Japanese ideographs) and conceptual stimuli appeared to induce a β-cognitive strategy (Francis and Irwin 1995; Irwin and Francis 1995). Yet, these were seen as exceptions. Although mere repetition of the same–different test did not appear to induce any change from the expected τ-strategy (Santosa and others 2010), requiring judges to assess the stimuli in the test separately, did bring about a change to a β-strategy for some judges (Lee and others 2007a). Further research (Chae and others 2010) indicated that various exercises designed to put judges in a various states of mind always induced a β-strategy. The old assumption of the automatic inducement of the τ-cognitive strategy has been challenged.

The picture for the various protocols of the A Not–A strategy is becoming established but needs more research. It can be argued that an assumption of a β- or a τ-cognitive strategy might depend on the nature of the exemplar in memory, to which a currently tasted food stimulus is being compared. Is it a short-lived exemplar elicited by tasting a prior standard? On the other hand, is it a relatively permanent exemplar which can be called upon once the experiment has ceased? This is worthy of investigation because it affects the choice of the cognitive strategy?

Research with ROC curves created the suspicion that different protocols for the A Not–A tests may not be equivalent. Presentation of a single standard (“A”) beforehand might induce a τ-strategy while presentation of more than one standard (“A” and “Not-A”) might induce a β-strategy. Then again, the A Not–AR might induce the τ-strategy (Lee and others 2007b, 2007c). Is the thoroughness of inspection when 2 standards are presented prior to the comparison stimuli, an important variable? Is the amount of warm-up (Meta-Garcia and others 2007) that might occur during the familiarization process, an important variable? Presumably they are important if they affect the type of memory exemplar available for comparison with later presented stimuli.

Previously, it had not been suspected that these changes in the protocol might actually change the test completely. This position has now been challenged. Yet, such a thing has happened before when Frijters (1979a) solved the so-called “paradox of discriminatory non-discriminators” (Byer and Abrams 1953; Gridgeman 1970). He used Thurstonian modeling to demonstrate that the 3-AFC and triangle tests were completely different; they used different cognitive strategies.

Psychophysicists interested in food and flavor stimuli, generally use judges who are not “expert” panelists and, as such, tend not to be over-familiar with the stimuli. Yet, in food science, expert panelists and consumers who are frequent consumers of a product are not a rarity. They may have exemplars in long-term memory that may induce β-cognitive strategies where τ-strategies might be expected. Again, this is a topic for future research because it concerns the relationship between expert panelists and untrained consumers.

A new technique is as good as its tools. The curve fitting tool for ROC curves used in the present Food Sensory Science experiments, would not have been possible without collaboration with Hautus (Lee and others 2007a, 2007b, 2007c; Hautus and others 2008, 2009; O'Mahony and Hautus 2008; Santosa and others 2010). This has initiated pioneering research and it is to be hoped that the models for fitting ROC curves will continue to develop. For the same–different test, a β- and τ-model are available. For the A Not–A test only a β-model is available. Accordingly, when the τ-model was applied to the A Not–A test in the experiments described previously, it was the τ-model for the same–different test. Yet, a τ-version of the A Not–A test is logically possible, where comparisons are made with an exemplar firmly embedded in memory using a τ-cognitive strategy.

The curve fitting in the experiments described previously dealt with a dichotomy, whether data were best fitted by a τ- or a β-cognitive strategy. Yet, are β- and τ-cognitive strategies the only ways that the brain can organize information while performing difference tests? Some of the experiments suggest alternative possible models. Novel cognitive strategies have been noted before (Tedja and others 1994). Santosa and others (2010) reported that for the same–different test, a judge reported using a “β-strategy for stimulus pairs.” This might have been illusory, but it is worth investigation. Lee and others (2007c) remarked that there was no strategy for A Not–A tests, where the reminder was presented as often as desired while tasting the comparison stimuli. There are obviously some questions to answer. Any new models should need to be able to produce ROC curves that can be distinguished from the classical τ- and β-models. However, modelers in this area are few and far between; more are needed, along with experimental research to test these models. Development of the right models are essential or else accurate d′ values cannot be computed for a certain class of difference tests.

Food sensory scientists deal with human behavioral responses, dependent on sensory input and the complexities of processing in the brain. They may be concerned with consumer acceptance, consumer perception, or using trained panelists to make analytical measurements of food attributes. Their “instruments” are generally human judges. The more they know about the “engineering” capabilities of their instrumentation, the more effective will be their measurements.
